# The Reverse Transcriptase Encoded by LINE-1 Retrotransposons in the Genesis, Progression, and Therapy of Cancer

**DOI:** 10.3389/fchem.2016.00006

**Published:** 2016-02-11

**Authors:** Ilaria Sciamanna, Chiara De Luca, Corrado Spadafora

**Affiliations:** ^1^Istituto Superiore di SanitàRome, Italy; ^2^Institute of Translational Pharmacology, National Resarch Council of ItalyRome, Italy

**Keywords:** LINE-1 retrotransposons, reverse transcriptase, tumorigenesis, differentiation therapy, cancer heterogeneity, epigenetics

## Abstract

In higher eukaryotic genomes, Long Interspersed Nuclear Element 1 (LINE-1) retrotransposons represent a large family of repeated genomic elements. They transpose using a reverse transcriptase (RT), which they encode as part of the ORF2p product. RT inhibition in cancer cells, either via RNA interference-dependent silencing of active LINE-1 elements, or using RT inhibitory drugs, reduces cancer cell proliferation, promotes their differentiation and antagonizes tumor progression in animal models. Indeed, the non-nucleoside RT inhibitor efavirenz has recently been tested in a phase II clinical trial with metastatic prostate cancer patients. An in-depth analysis of ORF2p in a mouse model of breast cancer showed ORF2p to be precociously expressed in precancerous lesions and highly abundant in advanced cancer stages, while being barely detectable in normal breast tissue, providing a rationale for the finding that RT-expressing tumors are therapeutically sensitive to RT inhibitors. We summarize mechanistic and gene profiling studies indicating that abundant LINE-1-derived RT can “sequester” RNA substrates for reverse transcription in tumor cells, entailing the formation of RNA:DNA hybrid molecules and impairing the overall production of regulatory miRNAs, with a global impact on the cell transcriptome. Based on these data, LINE-1-ORF2 encoded RT has a tumor-promoting potential that is exerted at an epigenetic level. We propose a model whereby LINE1-RT drives a previously unrecognized global regulatory process, the deregulation of which drives cell transformation and tumorigenesis with possible implications for cancer cell heterogeneity.

## Introduction: The retrotransposition machinery in the genesis of genomic and epigenomic landscapes

The complete sequencing of the human genome has disclosed the unexpected finding that coding genes account for a mere 1.2% of the total genome, while the remaining portion is constituted by non-coding DNA (International Human Genome Sequencing Consortium, 2001). Branded by a historically “bad reputation”, non-coding sequences have been defined as “junk” (Ohno, 1972) or “selfish” (Orgel and Crick, 1980) DNA, a view further strengthened by the evidence that nearly 50% of the human genome is constituted by apparently functionless transposable “genetic parasites” thought to increasingly litter all chromosomes during evolution.

Two main families of transposable elements characterize eukaryotic genomes: DNA transposons, which mobilize through a “cut and paste” mechanism (Muñoz-López and García-Pérez, 2010), and retrotransposons, which mobilize instead through “copy and paste,” a process that requires the reverse transcription of RNA intermediates into cDNA copies as a preliminary step in retrotransposition (Levin and Moran, 2011), promoting the broad expansion of retroelements in eukaryotic genomes.

A key player in this mechanism is the enzyme reverse transcriptase (RT) encoded by LINE-1 retrotransposons themselves. The latter are a source of the RT activity required to promote retrotransposition in human cells (Brouha et al., 2003). LINE-1 elements actually harbor two open reading frames, ORF1 and ORF2, which respectively encode ORF1p, an RNA-binding protein, and ORF2p, with reverse transcriptase (RT) and endonuclease (EN) activities (reviewed in Babushok and Kazazian, 2007). The LINE-1-derived retrotransposition machinery, constituted by ORF1 and ORF2 proteins, has *cis*-preference for its own LINE-1 RNA (Esnault et al., 2000; Wei et al., 2001; Kulpa and Moran, 2006). LINE-1-derived RT is also used for retrotranscription /retrotransposition of other RNAs, including Alu elements (Dewannieux et al., 2003), SVA (SINE-R/VNTR/ALU) elements (Ostertag et al., 2003) and mRNAs that give rise to a large population of processed pseudogenes nearly as numerous as the original coding genes (Pink et al., 2011). It is now well established that RT-originating sequences contribute to shape genomes and constitute a large proportion of evolutionarily conserved chromosomal DNA, accounting altogether for nearly 50% of the human genome (International Human Genome Sequencing Consortium, 2001). Such extensive preservation suggests a functional importance of retrotransposons. Not surprisingly, retrotransposons are increasingly being implicated in fundamental genomic functions, in both normal and pathological contexts (Rebollo et al., 2012). Indeed, as highly dynamic components of genomes, they contribute a relentless source of genetic and epigenetic variations and novelty (Feschotte, 2008; Bourque, 2009; Beck et al., 2011) and, on the long run, a major driving force in genome evolution (Oliver and Greene, 2011). A detailed description of all functional implications of retrotransposition in genome biology and evolution would be out of scope in this article, but extensive information is discussed in excellent reviews (Feschotte, 2008; Goodier and Kazazian, 2008; Bourque, 2009; Beck et al., 2011; Oliver and Greene, 2011; Rebollo et al., 2012).

The advent of high-throughput technologies in recent years has provided an accurate localization of new genomic insertions, shifting the focus from a gene-centric to a genome-wide view. This has revolutionized the traditional paradigms of genome organization by disclosing novel and unexpectedly complex genomic landscapes. Studies now show that genomes are crowded with sequences of reverse-transcribed origin, many of which are correlated with the insurgence of a variety of pathologies (for a review Hancks and Kazazian, 2012), in particular cancer (Belancio et al., 2010).

The ENCODE Project Consortium (The ENCODE Project Consortium, 2012) showed that approximately 80% of the human genome is pervasively transcribed; actually, a relevant proportion of small and long non-coding transcripts are functional components of genome-wide regulatory networks (Djebali et al., 2012). The groundbreaking finding of an astounding landscape of small RNAs—classified as microRNAs (miRNAs), endogenous small interfering RNAs (endo-siRNAs or siRNAs) (Piatek and Werner, 2014) and Piwi-interacting RNAs (piRNAs) (Kim et al., 2009), depending on their origin and the proteins they interact with - has unveiled an RNA-mediated regulatory network that controls the genome architecture and transcriptomic profile (Aalto and Pasquinelli, 2012; Li, 2014), influencing a multitude of biological processes. Growing data show a dual relationship between small RNAs and retroelements: on the one hand, small RNAs act as “guardians of the genome” in transposon-defense pathways aimed at repressing retroelement mobility (Yang and Kazazian, 2006; reviewed in Malone and Hannon, 2009); on the other hand, retroelements are intimately involved in their biogenesis, because a growing number of small RNAs in all three classes have a recognized retrotransposon-derived origin (Borchert et al., 2011; Watanabe et al., 2011).

Long non-coding RNA (lncRNAs) are components of the mammalian transcriptome and constitute a heterogeneous class of thousands of polymerase II-transcribed RNA species, polyadenylated, spliced, mostly localized in the nucleus (reviewed by Zhang et al., 2013). A large proportion of lncRNAs, with either oncogenic or tumor suppressor roles, are constituted by antisense RNAs; the latter, together with sense transcripts, are being identified in genome-wide regulatory networks that epigenetically fine-tune genome expression, with implications in tumorigenesis, differentiation and development (reviewed in Pelechano and Steinmetz, 2013, Fatica and Bozzoni, 2014). lncRNAs also have tight connections with transposable elements of both the DNA-based and the retroelements families, which occur within nearly 80% of mature lncRNA transcripts and account for about 30–40% of total human lncRNA sequences (Kelley and Rinn, 2012; Kapusta et al., 2013). Also of RT-derived origin are a large proportion of genomic sequences highly preserved throughout evolution and classified as conserved, highly-conserved and ultra-conserved elements (UCRs), according to the level of conservation throughout species (Bejerano et al., 2004; Woolfe et al., 2005, for a recent review see Nelson and Wardle, 2013).

From an ample survey encompassing the genomes of 29 mammalian species (Lowe and Haussler, 2012), a vision of genomes emerges as complex integrated functional systems, in which a considerable proportion of non-exonic sequences were exapted from mobile element insertions (Nishihara et al., 2006; Lowe and Haussler, 2012) to assemble large-scale regulatory circuits. Deregulation of these circuits is implicated in a variety of diseases, including cancer (Esteller, 2011).

## LINE-1-encoded RT as a new underestimated player in cancer

While retrotransposable elements are extensively studied and characterized, somewhat surprisingly the retrotransposon-encoded RT activity has long failed to attract an equivalent attention. The RT encoded by infective retroviruses has actually been intensively studied since the time of its discovery in 1970 by Baltimore (1970) and Temin and Mizutani (1970), due to its clinical implications (Herschhorn and Hizi, 2010). In contrast, the endogenous cellular RT has long been overlooked, despite many clues implicating it in as relevant processes as embryogenesis and tumorigenesis. Decades after the discoveries of Baltimore and Temin, a body of evidence has shown that endogenous RT expression is developmentally modulated: low levels of RT, if any, are expressed in differentiated non pathological tissues; increased expression is instead typical of cells characterized by low differentiation and high proliferation, e.g., early embryos (for a review see Sciamanna et al., 2011) and transformed cells (for a review see Sinibaldi-Vallebona et al., 2011). Overall, that is consistent with the notion that LINE-1 increased expression (Chen et al., 2012a; Rodić et al., 2014) and retroelement mobilization are implicated in tumorigenesis (Hancks and Kazazian, 2012; Kaer and Speek, 2013).

In contrast to differentiated quiescent cells, tissues and cells with low differentiation and high proliferation states are sites of high RT expression and provide permissive contexts for retrotransposition. Following up on that line, several studies have pursued RT inhibition in cancer cells, either using non-nucleoside RT inhibitors (nevirapine and efavirenz; Mangiacasale et al., 2003; Landriscina et al., 2005; Sciamanna et al., 2005, 2013), or RNA interference (RNAi)-mediated downregulation of RT-encoding LINE-1 elements (Sciamanna et al., 2005; Oricchio et al., 2007). In the latter case, the RNAi assays were carried out using double-stranded siRNA oligonucleotide targeted against the ORF-1 encoding domain of human full-length, highly expressed LINE-1s (Brouha et al., 2003). Both the drug-mediated and the RNAi-mediated approaches to reduce LINE-1-derived RT were found to reduce proliferation, promote differentiation and reprogram the global transcription profiles of coding and non-coding sequences in several cancer cell lines (human melanoma, glioblastoma, osteosarcoma and prostate, colon and small cell lung carcinomas). This provided early evidence for the implication of the LINE-1-encoded RT in tumorigenesis. The inhibitory effects of efavirenz on LINE-1 reverse transcription and retrotransposition were further tested in *in vitro* assays (Dai et al., 2011), and its antiproliferative and differentiating potential have been recently confirmed in breast (Patnala et al., 2013) and pancreatic (Hecht et al., 2015) cancer cell lines. Moreover, efavirenz treatment of mice xenografted with human tumorigenic cells caused the arrest, or a significant slow down, of progression of several tumor types *in vivo* (Sciamanna et al., 2005). Importantly, RNAi-mediated LINE-1 downregulation drastically reduced the tumorigenic potential of human cancer cells in nude mice (Oricchio et al., 2007). These effects are reversible and, upon discontinuation of RT inhibitory treatments, tumor cells return to their original de-differentiated phenotype and unrestrained proliferation capacity (Sciamanna et al., 2005); these obervations provided initial hints to an epigenetic role of RT.

The high levels of RT activity found in tumor cells and tissues, reported by our (Mangiacasale et al., 2003; Gualtieri et al., 2013) and other laboratories (Patnala et al., 2013), correlate well with the enhanced rate of retrotransposition observed in many human tumors, a phenomenon that dramatically contributes to shape cancer genomes (Iskow et al., 2010; Lee et al., 2012; Solyom et al., 2012; Shukla et al., 2013; Ewing et al., 2015). In a MMTV-PyVT transgenic mouse strain (Guy et al., 1992), whose females spontaneously develop breast carcinoma, a burst in the copy number of both LINE-1 and SINE B1 elements was depicted very early at tumor onset; their copy number further increases along with tumor progression (Gualtieri et al., 2013). These data converge to indicate that tumors constitute a highly permissive environment for retrotranscription, yet do not answer the question of whether overexpression and amplification of LINE-1 elements act as oncological “drivers” or as mere “passengers” (Rodic and Burns, 2013). The findings that pharmacological inhibition of RT is sufficient to reduce cancer cell proliferation, promote differentiation and antagonize tumor progression in animal models, similar to the effects obtained by RNAi-specific downregulation of LINE-1 expression, strongly support a causative role of LINE-1-encoded RT in tumorigenesis. In an applied clinical perspective, therefore, RT can be regarded as a target and RT inhibitors as potential therapeutic agents in a novel cancer differentiation therapy. Efavirenz has recently been tested in a phase II trial with metastatic prostate cancer patients, suggesting that relatively high dosage (over 600 mg per day) may be beneficial as a novel anticancer treatment (Houédé et al., 2014).

The role of RT encoded by LINE-1 in tumorigenesis is distinct from that of RT activities produced from the other two potential sources, i.e., endogenous retroviruses (HERVs) and telomerase-associated RT (TERT). First, RNAi-mediated downregulation of HERV-K expression showed negligible effects on the rate of proliferation and differentiation of cancer cells, in contrast with the dramatic effects observed after LINE-1-specific RNAi (Oricchio et al., 2007). Second, inhibitors of LINE-1 derived RT elicit rapid changes in treated cells in our experiments (Mangiacasale et al., 2003; Sciamanna et al., 2005), differently from drugs targeting telomerase, which reduce cancer cell proliferation after a long tratement (about 120 days; Damm et al., 2001); these data therefore rule out the possibility that TERT contributes to the rapid response of cells to RT inhibitors. It should be noted, however, that LINE-1 RT is critical for telomere maintenance, given that LINE-1 knockdown in cancer cells correlates with: (i) reduced length of telomeres, (ii) decreased telomerase activity, and (iii) decreased telomerase mRNA level (Aschacher et al., 2016). Together these results reveal that LINE-1 RT has a functional impact on TERT. Thus, while TERT is not involved in the changes elicited by inhibitors targeting the retrotransposon-derived genuine RT, the level of activity of LINE-1 elements may impact on TERT. These findings again strengthen the view that LINE-1 RT is a major player in tumorigenesis.

## LINE-1 ORF2-encoded RT activity in cancer progression

The ORF2-encoded RT has been recently assessed for its suitability as a tumor marker (Gualtieri et al., 2013) in females of the cancer-prone MMTV-PyVT described above (Guy et al., 1992). In these females, breast cancer tissues withdrawn at different times after birth are representative of progressive cancer stages. ORF2p cannot be detected in normal breast tissue by immunohistochemistry (IHC), but increased expression is triggered very early in tumorigenesis, preceding the appearance of typical histological alterations and accepted cancer markers (e.g., Ki67 and epidermal growth factor receptor ERB2); further upregulation takes place during tumor growth. These findings correlate well with the notion that hypomethylated LINE-1 sequences, from which ORF2p is produced, are typical of cancer genomes and precancerous lesions compared to their normal tissues counterpart (Miousse and Koturbash, 2015).

The abundant expression of LINE-1 products in preneoplastic mammary tissues suggests an exploitable tool as a potential diagnostic biomarker for early cancer detection: the identification of cancer-prone foci marked by increased RT before the appearance of recognizable histological alterations, can expand the window of opportunities for therapeutical intervention, which can possibly be most effective if associated with the development of RT inhibitory treatment. Interestingly, the abundance and subcellular localization of LINE-1 products are also proposed to have prognostic value in human metastatic breast cancer (Chen et al., 2012a).

Compelling objectives of “the war on cancer” currently include the definition of novel early markers identifying cancer-prone lesions before their spreading, as well as the development of novel therapeutic approaches in possible replacement of conventional cytotoxic chemotherapy. In a recent critical reappraisal, Hanahan (2014) has pointed out that the war on cancer, if not lost, is certainly not won and has suggested that therapeutic strategies should avoid fragmenting along multiple, highly diversified narrow paths targeting many substrates, each of which is highly selective for a specific cancer. Rather, the therapeutic “bullets” ought to hit fewer targets shared by a large spectrum of cancers (Hanahan, 2014). LINE-1 ORF2-encoded RT would fulfill these criteria, representing, at the same time, an early diagnostic cancer marker, a worth pursuing therapeutic target and the driving component of a newly emerging cancer-promoting mechanism.

## The molecular bases of the RT-dependent cancer-promoting mechanism

As briefly recalled above, retrotransposition events have had a fundamental role not only in shaping the genomic landscape, but also in directing regulatory networks aimed to fine-tune a variety of genomic functions. Data obtained in the last few years growingly indicate that the retrotransposon machinery, besides being a well-known source of genomic variations caused by new insertions (Böehne et al., 2008; Bourque, 2009), also exerts a global epigenetic regulatory role on the cellular transcriptome. LINE-1 ORF2-encoded RT is a new player in this mechanism.

Prompted by the finding that tumor cell lines are endowed with abundant LINE-1-encoded RT, Sciamanna et al. (2013) began to address the mechanism through which RT might act by comparing the global transcription profile of melanoma cells before and after RT inhibition by microarray analysis. The results showed that RT inhibition modulates the expression of a broad range of coding genes, but also long and small non-coding sequences, including UCRs and miRNAs. miRNAs actually emerged as crucial components of the RT-depending mechanism; indeed, a subpopulation, known to be involved in cell differentiation, cell growth, tumorigenesis and metastatic progression proved highly responsive to RT inhibition. Many miRNA-encoding genes are significantly associated with genomic regions enriched in closely spaced Alu repeats, further strengthening the link between miRNAs and retrotransposons. The physical association of pre-miRNA genomic loci with high density retroelements actually suggests that the latter can exert a regulatory “position effect” on miRNA expression (Slotkin and Martienssen, 2007). Experimental evidence supporting an orchestrating role of the RT enzyme emerged from cesium chloride density centrifugation analysis of nucleic acids extracted from melanoma and prostate carcinoma cell lines, harboring either “native” or efavirenz-inhibited RT: by buoyant density analysis, LINE-1- and Alu-containing molecules with the density of DNA:RNA hybrids were selectively identified in tumor cells, which disappeared upon treatment with efavirenz and were absent in non-transformed human fibroblasts (Sciamanna et al., 2013). Thus, the DNA:RNA hybrids are an especially abundant, if not exclusive, component of cancer cells, generated by reverse transcription of RNA templates, largely—albeit not exclusively—provided by LINE-1 and Alu transcripts. These data suggest that a cancer-promoting RT-dependent mechanism is active in tumor cells and can be blocked by inhibiting the LINE-1 RT. Based on these data, Sciamanna et al. proposed a model (Sciamanna et al., 2014) whereby the highly expressed LINE-1 RT in cancer cells can intercept RNAs and convert them in RNA:DNA hybrids via reverse transcription. Central to the model is the RT-dependent production of RNA:DNA hybrids, associated with altered functional miRNA profiles, observed under conditions of high LINE-1-derived RT in cancer cells and modulatable by RT inhibitors. A wealth of data show that miRNA expression is indeed downregulated in cancer cells, with profound implications for cell fates (Lu et al., 2005; Gaur et al., 2007; Jansson and Lund, 2012). A variety of small RNAs, including 7SL RNA (Ullu and Weiner, 1984), tRNAs (Kaçar et al., 1992), small nuclear RNAs (Doucet et al., 2015), and YRNAs (Perreault et al., 2005), are known to act as templates for reverse transcription in intermediate steps of the genesis of pseudogenes. It is not unreasonable to hypothesize that miRNA precursors may also be retrotranscribed. The observation that the production of hybrid RNA-DNA molecules is associated with aberrant miRNA profiles in cancer cells actually suggests that RT can “subtract” RNA precursors, thus preventing or impairing the formation of double stranded (ds) RNA dicer substrates for the biogenesis of mature miRNAs: this would ultimately contribute to establish favorable conditions for the onset of cancer phenotypes.

RT inhibition results in restored miRNA biogenesis, likely re-establishing their regulatory networks, consistent with its empirically established capacity to revert the cancer phenotype (Sciamanna et al., 2013).

In agreement with this idea, a subset of LINE-1-specific siRNAs, targeting LINE-1 expression and capable to induce methylation of their promoters, are found to be down-modulated in breast cancer compared to normal cells (Chen et al., 2012b). Conversely, LINE-1 inhibition by siRNAs up-modulate the expression of miRNAs involved in tumor suppression (Ohms et al., 2014). Taken as a whole, these findings indicate an orchestrating role of LINE-1-encoded RT in setting a cancer-permissive cellular state.

Although, the LINE-1 enzymatic machinery preferentially reverse transcribes its own RNA (Esnault et al., 2000; Wei et al., 2001; Kulpa and Moran, 2006), the presence of intronless pseudogenes scattered throughout mammalian genomes points out that mRNAs transcribed from protein-coding genes are also substrates for reverse transcription by the endogenous RT (Pink et al., 2011). This suggests that the RT-depending mechanism, in addition to targeting miRNAs, can also target several more RNA classes, coding and non-coding, small- and long-RNAs, though with a possible preferential bias for those associated with, or derived from, retroelement sequences. Consistent with this view, Sciamanna et al. (2013) found that about one third of the efavirenz-downmodulated miRNAs in melanoma cells are clustered on chromosome 19 (C19MC) in a locus characterized by a high density of primate-specific Alu repeats, which were shown to have co-evolved with miRNAs coding genes (Lehnert et al., 2009). An independent study also reported that LINE-1 silencing caused a deregulated profile of miRNA expression in breast cancer cells (Ohms and Rangasamy, 2014).

In summary, LINE-1 expression and small RNA networks emerge as the balanced components of a RT-depending regulatory mechanism placed at the intersection between normally differentiated and transformed non-differentiated cellular states: when one component raises the other one decays.

It is worth stressing that the partial inactivation of miRNA function is not an exclusive feature of cancer, but is a physiological phenomenon, shared with early preimplantation embryos, a context where again miRNA pathways become transiently suppressed (Suh et al., 2010). Moreover, miRNA inactivation is concomitant with a burst of LINE-1 activity in both tumorigenesis and embryogenesis. In the next paragraph we discuss this striking analogy and suggest that physiological and pathological processes have in common the same RT-dependent mechanism.

## The RT-based mechanism as global regulator of differentiation in tumorigenesis and embryogenesis

In prior developmental studies, the presence of LINE-1-encoded RT activity and protein was assessed in gametes and early embryos to address the potential role of this enzyme in embryogenesis. Unexpectedly, Giordano et al. (2000) found an RT activity in mature murine spermatozoa, providing the first hint that RT might somehow be involved in early embryogenesis. The sperm endogenous RT, far from being a nonfunctional remnant encoded by “genomic parasites,” has a full enzymatic activity able to reverse transcribe exogenous RNA molecules, taken up and internalized by spermatozoa, in cDNA copies that could then be delivered to embryos at fertilization (Giordano et al., 2000; reviewed in Spadafora, 2008). Pittoggi et al. (2003) further found that RT is also present in early embryos and is strictly required for preimplantation development: indeed, exposing zygotes to RT inhibitor, or antisense-mediated downregulation of LINE-1 (Beraldi et al., 2006), both caused a drastic arrest of development at the two- and four-cell embryo stages with globally altered gene expression profiles. Interestingly, fertilization activates a reverse transcription wave in zygotes within a few hours, which then propagates throughout the first cell division; that is concomitant with the production of new LINE-1 copies that mostly remain as non integrated extrachromosomal structures (Vitullo et al., 2012). Indirect evidence for an embryonic RT activity also emerge from reports that somatic LINE-1 retrotranspositions occur in human stem cells (Garcia-Perez et al., 2007; Coufal et al., 2009) and in very early stages of development in humans (van den Hurk et al., 2007) and rodents using transgenic murine and rat models (Kano et al., 2009). These findings indicate that the endogenous RT is active in early stages of embryogenesis, where it appears to have implications for epigenetic regulation of gene expression and to be necessary for the unfolding of the developmental program.

Cancer and embryo developmental studies convergingly point to the conclusion that an RT-based mechanism is physiologically activated in early embryogenesis and repressed in differentiated tissues; its unscheduled re-activation in somatic cells has cancer-promoting effects, yielding increased cell proliferation and loss of differentiation, in analogy with embryonic growth. It is a well-established notion that tumors and embryos share a variety of cellular, biochemical and molecular features and that genes typically expressed in embryogenesis, yet silenced in normal differentiated tissues, are re-expressed in tumors (Ma et al., 2010). These circumstances support the conclusion that tumorigenesis often recapitulates developmental patterns (Kaiser et al., 2007). In this conceptual framework, Spadafora (Spadafora, 2015) proposed that the RT-dependent mechanism is a source of the functional analogies shared by the physiological and pathological processes connecting embryogenesis and tumorigenesis.

## The genesis of cancer heterogeneity

**Figure 1:**
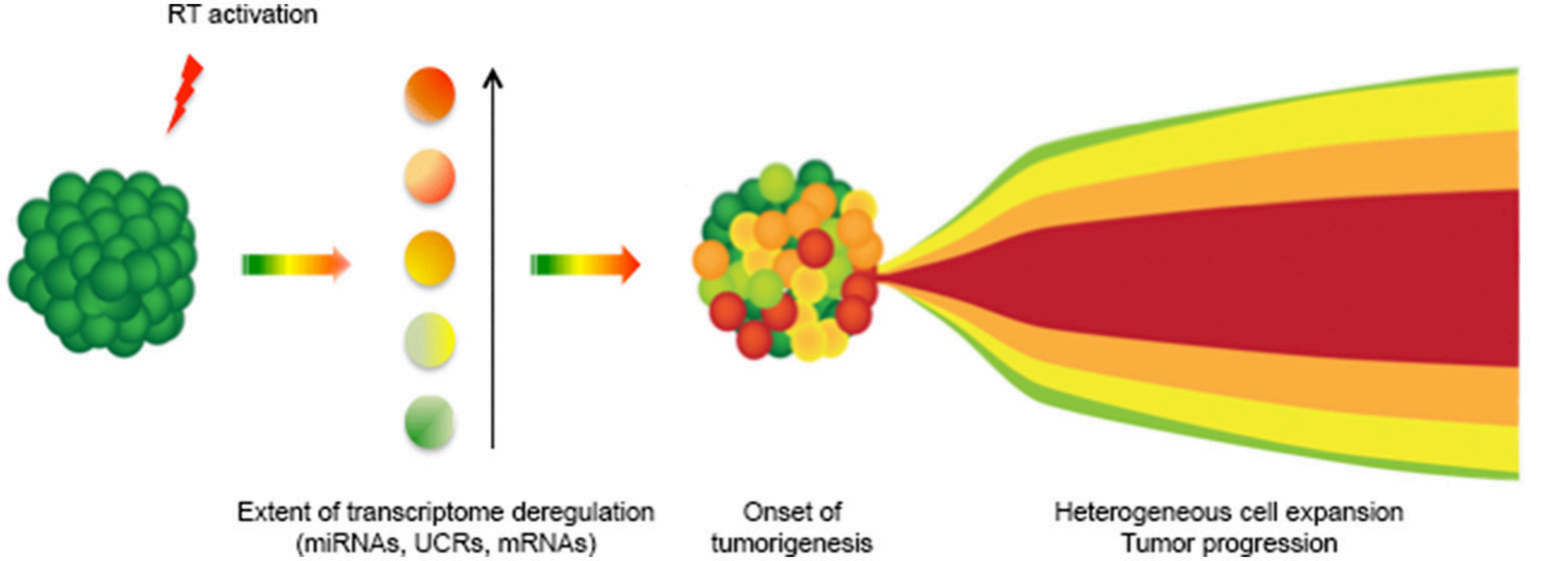
**A model for RT-dependent induction of cancer cell heterogeneity**. The deregulated expression of LINE-1 elements in somatic cells (green) causes a burst of RT activity (red flash), which deregulates the transcriptome of individual cells at various levels (represented by different color shades): this originates heterogeneous cancer cell populations. In the model, cancer cell heterogeneity would therefore set in following the early burst of differentially expressed RT activity in different cells. Cancer would then progress with the expansion of various cell populations (on the right).

The retrotransposon machinery is highly sensitive to stressing stimuli (Hagan and Rudin, 2002; Terasaki et al., 2013). In response, LINE-1 expression can be activated at differential levels in different cells, depending on the nature and the intensity of endogenous or exogenous stressors. We propose that the differential activation of RT, including by stress, can generate the heterogeneously differentiated cell populations that typically characterize human cancers (Meachem and Morrison, 2013). It is currently unclear whether the cellular heterogeneity observed in cancer reflects the existence of cell populations undergoing a progressive transformation “trajectory,” initiating as a primary cancer state and sequentially evolving into metastatic cells, or whether a broad array of cellular variations simultaneously arise in a single stress-responding event. Based on the data discussed above, it is tempting to speculate that the latter is the case; cells with varying degrees of malignancy—some of which may confer metastatic capacity—may concomitantly originate in a single genome-wide burst of stress-activated LINE-1-RT expression. In this hypothetical model, schematized in Figure 1, burst(s) of LINE-1 expression, triggered by exogenous and/or endogenous stimuli in normal cells (in green), would generate an array of cell populations endowed with various levels of LINE-1-dependent RT activity (indicated by different shades of colored cells), coinciding with the emergence of preneoplastic lesions. We propose that the different levels of LINE-1 activation correspond to different degrees of cell de-differentiation; in the process, embryonic regulatory patterns can be reactivated and induce somatic cells to revert back to embryo-like states (Kaiser et al., 2007). LINE-1 activation at low levels would exert modest de-defferentiation effects, while higher levels would determine a more extensive reactivation of embryonic patterns, with the ensuing production of more aggressive “embryo-like” transformed cells. The cell populations concomitantly originating from the activation of RT expression would then differentially propagate throughout cancer progression, thus contributing to cancer heterogeneity. The model represented in Figure 1 was inspired by the recently proposed “Big Bang” hypothesis for the genesis of human cancer, in which a single ancestral event is thought to originate the heterogeneity of cancer cell populations (Sottoriva et al., 2015), which would then progress and expand in parallel (Klein, 2009). The simultaneous genesis of cells with heterogeneous invasive potential would also offer a possible explanation for the genesis and spreading of metastatic tumors of unknown primary origin: these are a relatively rare class of metastatic tumors detected in patients in which the primary tumor cannot be identified, and account for 3–5% of all cancer diagnoses (Natoli et al., 2011; Stella et al., 2012).

In more general terms, the model predicts a relatively minor role for DNA mutations in cancer progression, as cell transformation is rather viewed as originating from an RT-mediated reactivation of “embryonic” regulatory circuits mostly acting at the epigenetic level in differentiated cells (Spadafora, 2015). Although needing further experimental testing, the model builds on emerging evidence indicating the global reach of RT onto several RNA classes (Sciamanna et al., 2013; Ohms et al., 2014; Ohms and Rangasamy, 2014) and is compatible with its reversible character by modulating RT levels (Sciamanna et al., 2005).

Retrotransposable elements also clearly impinge on genome function by generating extensive variations via insertional mutagenesis. Although large numbers of mutations are identified by high-throughput sequencing data in cancer contexts, their role(s) in tumorigenesis is often undefined (Kandoth et al., 2013): predisposing gene mutations in fact play a documented causative role only in 5–10% of human cancers (Nagy et al., 2004).

Recent excellent works have reported that the genomes of different tumor types harbor hundreds of *de novo* somatic insertions, selectively found in cancer genomes (Iskow et al., 2010; Lee et al., 2012; Solyom et al., 2012; Shukla et al., 2013; Doucet-O'Hare et al., 2015; Ewing et al., 2015; Rodić et al., 2015). Despite of these reports, however, the general implication of L1 retrotransposition events as either “driver” mutations (i.e., with a causative role in tumorigenesis), or as “passengers” (i.e., manifesting a consequence of the loss of genome regulation associated with cell transformation), remains an open question (Rodic and Burns, 2013). Insertions were documented and were attributed causative trigger in specific cases; among others, LINE-1 insertion were found within the *c-myc* gene (Morse et al., 1988), or in the tumor-suppressing gene apc (Miki et al., 1992) in breast and colon carcinoma, respectively; in those instances, LINE-1 insertions should have an activating (c-myc) or inhibitory (apc) role, respectively. In a different context, Alu insertions also result in neurofibromatosis type 1 (Wallace et al., 1991). It is worth recalling, however, that together LINE-1, Alu and SVA insertions account only for a marginal contribution (<0.5%) to the genesis of cancer (Callinan and Batzer, 2006). This leaves ample room for non-inserational mechanisms of tumorigenesis that implicate retrotransposons. In addition, the concept that new insertions might cause tumorigenesis would be hard to reconcile with the full reversibility of the “therapeutic” effect associated with LINE-1 RT inhibition in various cancer cells, observed in our and other laboratories (Sciamanna et al., 2005; Oricchio et al., 2007; Patnala et al., 2013). In our model, therefore, insertional mutagenesis, though not being totally ruled out, plays a minor role. We believe that most retrotranspositional insertions observed in many tumors reflect a failure to repress the activity of retroelements (a frequent failure in cancer), rather than being a cause of tumorigenesis.

In an extreme view, mutations may often represent a tolerated consequence of the tumor-associated global deregulation rather than the cause. The evidence summarized so far suggest that deregulated RT activity, likely acting in combination with other key epigenetic processes such as global DNA hypomethylation and chromatin remodeling, contributes to shape pro-tumorigenic expression profiles, and thus favors the phenotypic plasticity and diversity of cancer cells.

## Cancer as a reversible “developmental” disorder and differentiation therapy

As discussed above, the non-coding RNA profiles modulated by RT can globally regulate cell differentiation. Evidence is emerging that unscheduled reactivation of RT, as occurring in cancer cells, or its developmentally regulated repression, as in normal cells, are sufficient to promote cell de-differentiation or, on the contrary, stabilize the differentiated state, respectively. Tumorigenesis can be viewed as the erroneous resumption of genome-wide networks active in embryogenesis and silenced in adult life, and the differentiation process can be regarded as a sequence of transient and reversible cellular states in which RT activity is variably activated. According to this view, cancer would also be a reversible phenomenon and, as such, potentially modulatable by RT-inhibitory differentiation-inducing agents. The idea that the “normal” differentiation program can be restored to cancer cells, with the loss or attenuation of tumorigenic phenotype, has inspired much research and clinical work in the last decades. Perhaps the best known example is the development of retinoic acid-based differentiation therapy, successfully applied to treat acute promyelocytic leukemia (APML). Retinoic acid is a powerful morphogen and differentiating agent and has been the object of intense studies in the last decades, the outcome of which cannot be exhaustively discussed here (reviewed by Tang and Gudas, 2011). Other attempts to apply the same principle to solid tumors, however, have had more limited results so far (reviewed by Leszczyniecka et al., 2001; Cruz and Matushansky, 2012). The data obtained from *in vitro* assays, preclinical tests on animal models and a recent human trial, converge in viewing the LINE-1-encoded RT as an effective target for a non-cytotoxic, differentiation-inducing cancer therapy; RT inihibition appears to be the common condition sufficent to reverse tumorigenicity and restore differentiation to a wide variety of cancer cells.

## Conclusions

Growing data undermine the concept of terminal differentiation as a stably acquired condition, revealing that: (i) differentiation states should rather be viewed as transient conditions, and (ii) even in the presence of genomic alterations, epigenetics often wins over genetics (Lotem and Sachs, 2002). Epigenetic changes can effectively bypass the genetic alterations associated with, or caused by, tumorigenesis and reprogram gene expression profiles, reverting, or mitigating, the malignant phenotypes of cells. LINE-1-encoded RT is emerging as a key epigenetic regulator at the intersection between normal and pathological development. As such, the level of RT activity has the potential to shift the biological balance of cells in one or the other direction. In our view, these findings and emerging concepts, besides their clinical implications, fulfill the early prediction by Temin that endogenous RT activity plays roles both in normal development, as in embryogenesis, and in pathologies as such as cancer (Temin, 1971).

## Funding

Work in our laboratory was supported by the Italian Ministry of Health grant n. 15ONC/8 “Endogenous Reverse Transcriptase as a tumor marker and causative agent of tumor onset and progression,” to CS.

### Conflict of interest statement

The authors declare that the research was conducted in the absence of any commercial or financial relationships that could be construed as a potential conflict of interest.

## References

[B1] Aalto A. P. Pasquinelli A. E. (2012). Small non-coding RNAs mount a silent revolution in gene expression. Curr. Opin. Cell Biol. 24, 333–340. 10.1016/j.ceb.2012.03.006 22464106 PMC3372702

[B2] Aschacher T. Wolf B. Enzmann F. Kienzl P. Messner B. Sampl S.. (2016). LINE-1 induces hTERT and ensures telomere maintenance in tumour cell lines. Oncogene 35, 94–104. 10.1038/onc.2015.65 25798839

[B3] Babushok D. V. Kazazian H. H. Jr. (2007). Progress in understanding the biology of the human mutagen LINE-1. Hum. Mutat. 28, 527–539. 10.1002/humu.20486 17309057

[B4] Baltimore D. (1970). RNA-dependent DNA polymerase in virions of RNA tumor viruses. Nature 226, 1209–1211. 10.1038/2261209a0 4316300

[B5] Beck C. R. Garcia-Perez J. L. Badge R. M. Moran J. V. (2011). LINE-1 elements in structural variation and disease. Annu. Rev. Genomics Hum. Genet. 12, 187–215. 10.1146/annurev-genom-082509-141802 21801021 PMC4124830

[B6] Bejerano G. Pheasant M. Makunin I. Stephen S. Kent W. J. Mattick J. S.. (2004). Ultraconserved elements in the human genome. Science 304, 1321–1325. 10.1126/science.1098119 15131266

[B7] Belancio V. P. Roy-Engel A. M. Deininger P. L. (2010). All y'all need to know'bout retroelements in cancer. Semin. Cancer Biol. 20, 200–210. 10.1016/j.semcancer.2010.06.001 20600922 PMC2943028

[B8] Beraldi R. Pittoggi C. Sciamanna I. Mattei E. Spadafora C. (2006). Expression of LINE-1 retroposons is essential for murine preimplantation development. Mol. Reprod. Dev. 73, 279–287. 10.1002/mrd.20423 16365895

[B9] Böehne A. Brunet F. Galiana-Arnoux D. Schultheis C. Volff J. N. (2008). Transposable elements as drivers of genomic and biological diversity in vertebrates. Chromosome Res. 16, 203–215. 10.1007/s10577-007-1202-6 18293113

[B10] Borchert G. M. Holton N. W. Williams J. D. Hernan W. L. Bishop I. P. Dembosky J. A.. (2011). Comprehensive analysis of microRNA genomic loci identifies pervasive repetitive-element origins. Mob. Genet. Elements 1, 8–17. 10.4161/mge.1.1.15766 22016841 PMC3190270

[B11] Bourque G. (2009). Transposable elements in gene regulation and in the evolution of vertebrate genomes. Curr. Opin. Genet. Dev. 19, 607–612. 10.1016/j.gde.2009.10.013 19914058

[B12] Brouha B. Schustak J. Badge R. M. Lutz-Prigge S. Farley A. H. Moran J. V.. (2003). Hot L1s account for the bulk of retrotransposition in the human population. Proc. Natl. Acad. Sci. U.S.A. 100, 5280–5285. 10.1073/pnas.0831042100 12682288 PMC154336

[B13] Callinan P. A. Batzer M. A. (2006). Retrotransposable elements and human disease. Genome Dyn. 1, 104–115. 10.1159/000092503 18724056

[B14] Chen L. Dahlstrom J. E. Chandra A. Board P. Rangasamy D. (2012a). Prognostic value of LINE-1 retrotransposon expression and its subcellular localization in breast cancer. Breast Cancer Res. Treat. 136, 129–142. 10.1007/s10549-012-2246-7 23053642 PMC3473189

[B15] Chen L. Dahlstrom J. E. Lee S. H. Rangasamy D. (2012b). Naturally occurring endo-siRNA silences LINE-1 retrotransposons in human cells through DNA methylation. Epigenetics 7, 758–771. 10.4161/epi.20706 22647391

[B16] Coufal N. G. Garcia-Perez J. L. Peng G. E. Yeo G. W. Mu Y. Lovci M. T.. (2009). L1 retrotransposition in human neural progenitor cells. Nature 460, 1127–1131. 10.1038/nature08248 19657334 PMC2909034

[B17] Cruz F. D. Matushansky I. (2012). Solid tumor differentiation therapy - is it possible? Oncotarget 3, 559–567. 10.18632/oncotarget.512 22643847 PMC3388185

[B18] Dai L. Huang Q. Boeke J. D. (2011). Effect of reverse transcriptase inhibitors on LINE-1 and Ty1 reverse transcriptase activities and on LINE-1 retrotransposition. BMC Biochem. 2:18. 10.1186/1471-2091-12-18 21545744 PMC3103432

[B19] Damm K. Hemmann U. Garin-Chesa P. Hauel N. Kauffmann I. Priepke H.. (2001). A highly selective telomerase inhibitor limiting human cancer cell proliferation. EMBO J. 20, 6958–6968. 10.1093/emboj/20.24.6958 11742973 PMC125790

[B20] Dewannieux M. Esnault C. Heidmann T. (2003). LINE-mediated retrotransposition of marked Alu sequences. Nat. Genet. 35, 41–48. 10.1038/ng1223 12897783

[B21] Djebali S. Davis C. A. Merkel A. Dobin A. Lassmann T. Mortazavi A.. (2012). Landscape of transcription in human cells. Nature 489, 101–108. 10.1038/nature11233 22955620 PMC3684276

[B22] Doucet A. J. Droc G. Siol O. Audoux J. Gilbert N. (2015). U6 snRNA Pseudogenes: markers of retrotransposition dynamics in mammals. Mol. Biol. Evol. 32, 1815–1832. 10.1093/molbev/msv062 25761766 PMC4476161

[B23] Doucet-O'Hare T. T. Rodic N. Sharma R. Darbari I. Abril G. Choi J. A.. (2015). LINE-1 expression and retrotransposition in Barrett's esophagus and esophageal carcinoma. Proc. Natl. Acad. Sci. U.S.A. 112, E4894–E900. 10.1073/pnas.1502474112 26283398 PMC4568228

[B24] Esnault C. Maestre J. Heidmann T. (2000). Human LINE retrotransposons generate processed pseudogenes. Nat. Genet. 24, 363–367. 10.1038/74184 10742098

[B25] Esteller M. (2011). Non-coding RNAs in human disease. Nat. Rev. Genet. 12, 861–874. 10.1038/nrg3074 22094949

[B26] Ewing A. D. Gacita A. Wood L. D. Ma F. Xing D. Kim M. S.. (2015). Widespread somatic L1 retrotransposition occurs early during gastrointestinal cancer evolution. Genome Res. 25, 1536–1545. 10.1101/gr.196238.115 26260970 PMC4579339

[B27] Fatica A. Bozzoni I. (2014). Long non-coding RNAs: new players in cell differentiation and development. Nat. Rev. 15, 7–21. 10.1038/nrg3606 24296535

[B28] Feschotte C. (2008). Transposable elements and the evolution of regulatory networks. Nat Rev. Genet. 9, 397–405. 10.1038/nrg2337 18368054 PMC2596197

[B29] Garcia-Perez J. L. Marchetto M. C. N. Muotri A. R. Coufal N. C. Gage F. H. O'shea K. S.. (2007). LINE-1 retrotransposition in human embryonic stem cells. Hum. Mol. Genet. 16, 1569–1577. 10.1093/hmg/ddm105 17468180

[B30] Gaur A. Jewell D. A. Liang Y. Ridzon D. Moore J. H. Chen C. (2007). Characterization of microRNA expression levels and their biological correlates in human cancer cell lines. Cancer Res. 67, 2456–2468. 10.1158/0008-5472.CAN-06-2698 17363563

[B31] Giordano R. Magnano A. R. Zaccagnini G. Pittoggi C. Moscufo N. Lorenzini R.. (2000). Reverse transcriptase activity in mature spermatozoa of mouse. J. Cell Biol. 148, 1107–1113. 10.1083/jcb.148.6.1107 10725323 PMC2174319

[B32] Goodier J. L. Kazazian H. H. Jr. (2008). Retrotransposons revisited: the restraint and rehabilitation of parasites. Cell 135, 23–35. 10.1016/j.cell.2008.09.022 18854152

[B33] Gualtieri A. Andreola F. Sciamanna I. Sinibaldi- Vallebona P. Serafino A. Spadafora C. (2013). Increased expression and copy number amplification of LINE-1 and SINE B1 retrotransposable elements in murine mammary carcinoma progression. Oncotarget 4, 1882–1893. 10.18632/oncotarget.1188 24231191 PMC3875756

[B34] Guy C. T. Cardiff R. D. Muller W. J. (1992). Induction of mammary tumors by expression of polyomavirus middle T oncogene: a transgenic mouse model for metastatic disease. Mol. Cell. Biol. 12, 954–961. 10.1128/MCB.12.3.954 1312220 PMC369527

[B35] Hagan C. R. Rudin C. M. (2002). Mobile genetic element activation and genotoxic cancer therapy potential clinical implications. Am. J. Pharmacogenomics 2, 25–35. 10.2165/00129785-200202010-00003 12083952

[B36] Hanahan D. (2014). Rethinking the war on cancer. Lancet 383, 558–563. 10.1016/S0140-6736(13)62226-6 24351321

[B37] Hancks D. C. Kazazian H. H. Jr. (2012). Active human retrotransposons: variation and disease. Curr. Opin. Genet. Dev. 22, 191–203. 10.1016/j.gde.2012.02.006 22406018 PMC3376660

[B38] Hecht M. Erber S. Harrer T. Klinker H. Roth T. Parsch H.. (2015). Efavirenz has the highest anti-proliferative effect of non-nucleoside reverse transcriptase pnhibitors against pancreatic cancer cells. PLoS ONE 10:e0130277. 10.1371/journal.pone.0130277 26086472 PMC4473268

[B39] Herschhorn A. Hizi A. (2010). Retroviral reverse transcriptases. Cell. Mol. Life Sci. 67, 2717–2747. 10.1007/s00018-010-0346-2 20358252 PMC11115783

[B40] Houédé N. Pulido M. Mourey L. Joly F. Ferrero J. M. Bellera C.. (2014). A phase II trial evaluating the efficacy and safety of efavirenz in metastatic castration-resistant prostate cancer. Oncologist 19, 1227–1228. 10.1634/theoncologist.2014-0345 25355844 PMC4257751

[B41] International Human Genome Sequencing Consortium (2001). Initial sequencing and analysis of the human genome. Nature 409, 860–921. 10.1038/35057062 11237011

[B42] Iskow R. C. McCabe M. T. Mills R. E. Torene S. Pittard W. S. Neuwald A. F.. (2010). Natural mutagenesis of human genomes by endogenous retrotransposons. Cell 141, 1253–1261. 10.1016/j.cell.2010.05.020 20603005 PMC2943760

[B43] Jansson M. D. Lund A. H. (2012). MicroRNA and cancer. Mol. Oncol. 6, 590–610. 10.1016/j.molonc.2012.09.006 23102669 PMC5528350

[B44] Kaçar Y. Thomann H. U. Gross H. J. (1992). The first human genes for tRNA(ArgICG), tRNA(GlyUCC), and tRNA(ThrIGU) and more tRNA(Val) pseudogenes: expression and pre-tRNA maturation in HeLa cell-free extracts. DNA Cell Biol. 11, 781–790. 10.1089/dna.1992.11.781 1457046

[B45] Kaer K. Speek M. (2013). Retroelements in human disease. Gene 518, 231–241. 10.1016/j.gene.2013.01.008 23333607

[B46] Kaiser S. Park Y. K. Franklin J. L. Halberg R. B. Yu M. Jessen W. J.. (2007). Transcriptional recapitulation and subversion of embryonic colon development by mouse colon tumor models and human colon cancer. Genome Biol. 8:R131. 10.1186/gb-2007-8-7-r131 17615082 PMC2323222

[B47] Kandoth C. McLellan M. D. Vandin F. Ye K. Niu B. Lu C.. (2013). Mutational landscape and significance across 12 major cancer types. Nature 502, 333–339. 10.1038/nature12634 24132290 PMC3927368

[B48] Kano H. Godoy I. Courtney C. Vetter M. R. Gerton G. L. Ostertag E. M.. (2009). L1 retrotransposition occurs mainly in embryogenesis and creates somatic mosaicism. Genes Dev. 23, 1303–1312. 10.1101/gad.1803909 19487571 PMC2701581

[B49] Kapusta A. Kronenberg Z. Lynch V. J. Zhuo X. Ramsay L. A. Bourque G.. (2013). Transposable elements are major contributors to the origin, diversification, and regulation of vertebrate long noncoding RNAs. PLoS Genet. 9:e1003470. 10.1371/journal.pgen.1003470 23637635 PMC3636048

[B50] Kelley D. Rinn J. (2012). Transposable elements reveal a stem cell-specific class of long noncoding RNAs. Genome Biol. 13:R107. 10.1186/gb-2012-13-11-r107 23181609 PMC3580499

[B51] Kim V. N. Han J. Siomi M. C. (2009). Biogenesis of small RNAs in animals. Nat. Rev. Mol. Cell Biol. 10, 126–139. 10.1038/nrm2632 19165215

[B52] Klein C. A. (2009). Parallel progression of primary tumours and metastases. Nat. Rev. Cancer 9, 302–312. 10.1038/nrc2627 19308069

[B53] Kulpa D. A. Moran J. V. (2006). Cis-preferential LINE-1 reverse transcriptase activity in ribonucleoprotein particles. Nat. Struc. Mol. Biol. 13, 655–660. 10.1038/nsmb1107 16783376

[B54] Landriscina M. Fabiano A. Altamura S. Bagala C. Piscazzi A. Cassano A.. (2005). Reverse transcriptase inhibitors downregulate cell proliferation *in vitro* and *in vivo* and restore TSH signaling and iodine uptake in human thyroid anaplastic carcinoma. J. Clin. Endocrinol. Metab. 90, 5663–5671. 10.1210/jc.2005-0367 16030158

[B55] Lee E. Iskow R. Yang L. Gokcumen O. Haseley P. Luquette L. J. III.. (2012). Landscape of somatic retrotransposition in human cancers. Science 337, 967–971. 10.1126/science.1222077 22745252 PMC3656569

[B56] Lehnert S. Van Loo P. Thilakarathne P. J. Marynen P. Verbeke G. Schuit F. (2009). Evidence for co-evolution between human microRNAs and Alu-repeats. PLoS ONE 4:e4456. 10.1371/journal.pone.0004456 19209240 PMC2637760

[B57] Leszczyniecka M. Roberts T. Dent P. Grant S. Fisher P. B. (2001). Differentiation therapy of human cancer: basic science and clinical applications. Pharmacol. Ther. 90, 105–196. 10.1016/S0163-7258(01)00132-2 11578655

[B58] Levin H. L. Moran J. V. (2011). Dynamic interactions between transposable elements and their hosts. Nat. Rev. Genet. 12, 615–627. 10.1038/nrg3030 21850042 PMC3192332

[B59] Li L. C. (2014). Chromatin remodeling by the small RNA machinery in mammalian cells. Epigenetics 9, 1–8. 10.4161/epi.26830 24149777 PMC3928185

[B60] Lotem J. Sachs L. (2002). Epigenetics wins over genetics: induction of differentiation in tumor cells. Semin. Cancer Biol. 12, 339–346. 10.1016/S1044-579X(02)00054-8 12191633

[B61] Lowe C. B. Haussler D. (2012). 29 Mammalian genomes reveal novel exaptations of mobile elements for likely regulatory functions in the human genome. PLoS ONE 7:e43128. 10.1371/journal.pone.0043128 22952639 PMC3428314

[B62] Lu J. Getz G. Miska E. A. Alvarez-Saavedra E. Lamb J. Peck D.. (2005). MicroRNA expression profiles classify human cancers. Nature 435, 834–838. 10.1038/nature03702 15944708

[B63] Ma Y. Zhang P. Wang F. Yang J. Yang Z. Qin H. (2010). The relationship between early embryo development and tumourigenesis. J. Cell. Mol. Med. 14, 2697–2701. 10.1111/j.1582-4934.2010.01191.x 21029369 PMC3822720

[B64] Malone C. D. Hannon G. J. (2009). Small RNAs as guardians of the genome. Cell 136, 656–668. 10.1016/j.cell.2009.01.045 19239887 PMC2792755

[B65] Mangiacasale R. Pittoggi C. Sciamanna I. Careddu A. Mattei E. Lorenzini R.. (2003). Exposure of normal and transformed cells to nevirapine, a reverse transcriptase inhibitor, reduces cell growth and promotes differentiation. Oncogene 22, 2750–2761. 10.1038/sj.onc.1206354 12747369

[B66] Meachem C. E. Morrison S. J. (2013). Tumour heterogeneity and cancer cell plasticity. Nature 501, 328–337. 10.1038/nature12624 24048065 PMC4521623

[B67] Miki Y. Nishisho I. Horii A. Miyoshi Y. Utsunomiya J. Kinzler K. W.. (1992). Disruption of the APC gene by a retrotransposal insertion of L1 sequence in a colon cancer. Cancer Res. 52, 643–645. 1310068

[B68] Miousse I. R. Koturbash I. (2015). The Fine LINE: methylation drawing the cancer landscape. Biomed Res. Int. 2015:131547. 10.1155/2015/131547 26448926 PMC4584040

[B69] Morse B. Rotherg P. G. South V. J. Spandorfer J. M. Astrin S. M. (1988). Insertional mutagenesis of the myc locus by a LINE-1 sequence in a hu- man breast carcinoma. Nature 333, 87–90. 10.1038/333087a0 2834650

[B70] Muñoz-López M. García-Pérez J. L. (2010). DNA Transposons: Nature and application in Genomics. Curr. Biol. 11, 115–128. 10.2174/138920210790886871 PMC2874221 20885819

[B71] Nagy R. Sweet K. Eng C. (2004). Highly penetrant hereditary cancer syndromes. Oncogene 23, 6445–6470. 10.1038/sj.onc.1207714 15322516

[B72] Natoli C. Ramazzotti V. Nappi O. Giacomini P. Palmeri S. Salvatore M.. (2011). Unknown primary tumors. Biochim. Biophys. Acta 1816, 13–24. 10.1016/j.bbcan.2011.02.002 21371531

[B73] Nelson A. C. Wardle F. C. (2013). Conserved non-coding elements and *cis* regulation: actions speak louder than words. Development 140, 1385–1395. 10.1242/dev.084459 23482485

[B74] Nishihara H. Smit A. F. A. Okada N. (2006). Functional noncoding sequences derived from SINEs in the mammalian genome. Genome Res. 16, 864–874. 10.1101/gr.5255506 16717141 PMC1484453

[B75] Ohms S. Lee S. H. Rangasamy D. (2014). LINE-1 retrotransposons and *let-7* miRNA: partners in the pathogenesis of cancer? Front Genet. 5:338 10.3389/fgene.2014.00338 PMC4188135 25339972

[B76] Ohms S. Rangasamy D. (2014). Silencing of LINE-1 retrotransposons contributes to variation in small noncoding RNA expression in human cancer cells. Oncotarget 5, 4103–4117. 10.18632/oncotarget.1822 24980824 PMC4147309

[B77] Ohno S. (1972). So much “junk” DNA in our genome. Brookhaven Symp. Biol. 23, 366–370. 5065367

[B78] Oliver K. R. Greene W. K. (2011). Mobile DNA and the TE-Thrust hypothesis: supporting evidence from the primates. Mob. DNA 2:8. 10.1186/1759-8753-2-8 21627776 PMC3123540

[B79] Orgel L. E. Crick F. H. (1980). Selfish DNA: the ultimate parasite. Nature 284, 604–607. 10.1038/284604a0 7366731

[B80] Oricchio E. Beraldi R. Sciamanna I. Tolstonog G. V. Schumann G. G. Spadafora C. (2007). Distinct roles for LINE-1 and Herv-K retroelements in cell proliferation, differentiation and tumor progression. Oncogene 26, 4226–4233. 10.1038/sj.onc.1210214 17237820

[B81] Ostertag E. M. Goodier J. L. Zhang Y. Kazazain H. H. Jr. (2003). SVA elements are nonautonomous retrotransposons that cause disease in humans. Am. J. Hum. Genet. 73, 1444–1451. 10.1086/380207 14628287 PMC1180407

[B82] Patnala R. Lee S. H. Dahlstrom J. E. Ohms S. Chen L. Dheen S. T.. (2013). Inhibition of LINE-1 retrotransposon-encoded reverse transcriptase modulates the expression of cell differentiation genes in breast cancer cells. Breast. Cancer Res. Treat 143, 239–253. 10.1007/s10549-013-2812-7 24337508 PMC3889873

[B83] Pelechano V. Steinmetz L. M. (2013). Gene regulation by antisense transcription. Nat Rev. 14, 880–893. 10.1038/nrg3594 24217315

[B84] Perreault J. Noël J. F. Brière F. Cousineau B. Lucier J. F.. (2005). Retropseudogenes derived from the human Ro/SS-A autoantigen-associated hY RNAs. Nucleic Acids Res. 33, 2032–2041. 10.1093/nar/gki504 15817567 PMC1074747

[B85] Piatek M. J. Werner A. (2014). Endogenous siRNAs: regulators of internal affairs. Biochem. Soc. Trans. 42, 1174–1179. 10.1042/BST20140068 25110021 PMC4289821

[B86] Pink R. C. Wicks K. Caley D. P. Punch E. K. Jacobs L. Carter D. R. F. (2011). Pseudogenes: Pseudo-functional or key regulators in health and disease? RNA 17, 792–798. 10.1261/rna.2658311 21398401 PMC3078729

[B87] Pittoggi C. Sciamanna I. Mattei E. Beraldi R. Lobascio A. M. Mai A.. (2003). Role of endogenous reverse transcriptase in murine early embryo development. Mol. Reprod. Dev. 66, 225–236. 10.1002/mrd.10349 14502601

[B88] Rebollo R. Romanish M. T. Mager D. L. (2012). Transposable elements: an abundant and natural source of regulatory sequences for host genes. Annu. Rev. Genet. 46, 21–42. 10.1146/annurev-genet-110711-155621 22905872

[B89] Rodic N. Burns K. H. (2013). Long interspersed element–1 (LINE-1): passenger or driver in human neoplasms? PLoS Genet. 9:e1003402. 10.1371/journal.pgen.1003402 23555307 PMC3610623

[B90] Rodić N. Sharma R. Sharma R. Zampella J. Dai L. Taylor M. S.. (2014). Long Interspersed Element-1 protein expression is a hallmark of many human cancers. Am. J. Pathol. 184, 1280–1286. 10.1016/j.ajpath.2014.01.007 24607009 PMC4005969

[B91] Rodić N. Steranka J. P. Makohon-Moore A. Moyer A. Shen P. Sharma R.. (2015). Retrotransposon insertions in the clonal evolution of pancreatic ductal adenocarcinoma. Nat. Med. 21, 1060–1064. 10.1038/nm.3919 26259033 PMC4775273

[B92] Sciamanna I. Gualtieri A. Cossetti C. Osimo E. F. Ferracin M. Macchia G.. (2013). A tumor-promoting mechanism mediated by retrotransposon- encoded reverse transcriptase is active in human transformed cell lines. Oncotarget 4, 2271–2287. 10.18632/oncotarget.1403 24345856 PMC3926826

[B93] Sciamanna I. Gualtieri A. Piazza P. V. Spadafora C. (2014). Regulatory roles of LINE-1-encoded reverse transcriptase in cancer onset and progression. Oncotarget 5, 8039–8051. 10.18632/oncotarget.2504 25478632 PMC4226666

[B94] Sciamanna I. Landriscina M. Pittoggi C. Quirino M. Marelli C. Beraldi R.. (2005). Inhibition of endogenous reverse transcriptase antagonizes human tumor growth. Oncogene 24, 3923–3931. 10.1038/sj.onc.1208562 15806170

[B95] Sciamanna I. Vitullo P. Curatolo A. Spadafora C. (2011). A reverse transcriptase-dependent mechanism is essential for murine preimplantation development. Genes 2, 360–373. 10.3390/genes2020360 24710196 PMC3924816

[B96] Shukla R. Upton K. R. Muñoz-Lopez M. Gerhardt D. J. Fisher M. E. Nguyen T.. (2013). Endogenous retrotransposition activates oncogenic pathways in hepatocellular carcinoma. Cell 153, 101–111. 10.1016/j.cell.2013.02.032 23540693 PMC3898742

[B97] Sinibaldi-Vallebona P. Matteucci C. Spadafora C. (2011). Retrotransposon-encoded reverse transcriptase in the genesis, progression and cellular plasticity of human cancer. Cancers 3, 1141–1157. 10.3390/cancers3011141 24212657 PMC3756407

[B98] Slotkin R. K. Martienssen R. (2007). Transposable elements and the epigenetic regulation of the genome. Nat. Rev. Genet. 8, 272–285. 10.1038/nrg2072 17363976

[B99] Solyom S. Ewing A. D. Rahrmann E. P. Doucet T. Nelson H. H. Burns M. B.. (2012). Extensive somatic L1 retrotransposition in colorectal tumors. Genome Res. 22, 2328–2338. 10.1101/gr.145235.112 22968929 PMC3514663

[B100] Sottoriva A. Kang H. Ma Z. Graham T. A. Salomon M. P. Zhao J.. (2015). A Big Bang model of human colorectal tumor growth. Nat. Genet. 47, 209–216. 10.1038/ng.3214 25665006 PMC4575589

[B101] Spadafora C. (2008). Sperm-mediated ‘reverse’ gene transfer: a role of reverse transcriptase in the generation of new genetic information. Hum. Reprod. 23, 735–740. 10.1093/humrep/dem425 18270183

[B102] Spadafora C. (2015). A LINE-1–encoded reverse transcriptase–dependent regulatory mechanism is active in embryogenesis and tumorigenesis. Ann. N.Y. Acad. Sci. 1341, 164–171. 10.1111/nyas.12637 25586649

[B103] Stella G. M. Senetta R. Cassenti A. Ronco M. Cassoni P. (2012). Cancers of unknown primary origin: current perspectives and future therapeutic strategies. J. Translat. Med. 10:12. 10.1186/1479-5876-10-12 22272606 PMC3315427

[B104] Suh N. Baehner L. Moltzahn F. Melton C. Shenoy A. Chen J.. (2010). MicroRNA function is globally suppressed in mouse oocytes and early embryos. Curr. Biol. 20, 271–277. 10.1016/j.cub.2009.12.044 20116247 PMC2872512

[B105] Tang X. H. Gudas L. J. (2011). Retinoids, retinoic acid receptors, and cancer. Annu. Rev. Pathol. 6, 345–364. 10.1146/annurev-pathol-011110-130303 21073338

[B106] Temin H. M. (1971). Guest editorial. The protovirus hypothesis: speculations on the significance of RNA-directed DNA synthesis for normal development and for carcinogenesis. J. Natl. Cancer Inst. 46, III–VI. 5115908

[B107] Temin H. M. Mizutani S. (1970). RNA-dependent DNA polymerase in virions of Rous sarcoma virus. Nature 226, 1211–1213. 10.1038/2261211a0 4316301

[B108] Terasaki N. Goodier J. L. Cheung L. E. Wang Y. J. Kajikawa M. Kazazian H. H. Jr.. (2013). *In vitro* screening for compounds that enhance human L1 mobilization. PLoS ONE 8:e74629. 10.1371/journal.pone.0074629 24040300 PMC3770661

[B109] The ENCODE Project Consortium (2012). An integrated encyclopedia of DNA elements in the human genome. Nature 489, 57–74. 10.1038/nature11247 22955616 PMC3439153

[B110] Ullu E. Weiner A. M. (1984). Human genes and pseudogenes for the 7SL RNA component of signal recognition particle. EMBO J. 3, 3303–3310 6084597 10.1002/j.1460-2075.1984.tb02294.x PMC557853

[B111] van den Hurk J. A. J. M. Meij I. C. Seleme M. C. Kano H. Nikopoulos K. Hoefsloot L. H.. (2007). L1 retrotransposition can occur early in human embryonic development. Hum. Mol. Genet. 16, 1587–1592. 10.1093/hmg/ddm108 17483097

[B112] Vitullo P. Sciamanna I. Baiocchi M. Sinibaldi-Vallebona P. Spadafora C. (2012). LINE-1 retrotransposon copies are amplified during murine early embryo development. Mol. Reprod. Dev. 79, 118–127. 10.1002/mrd.22003 22139884

[B113] Wallace M. R. Andersen L. B. Saulino A. M. Gregory P. E. Glover T. W. Collins F. S. (1991). A *de novo Alu* insertion results in neurofibromatosis type 1. Nature 353, 864–866. 10.1038/353864a0 1719426

[B114] Watanabe T. Tomizawa S. Mitsuya K. Totoki Y. Yamamoto Y.. (2011). Role for piRNAs and noncoding RNA in de novo DNA methylation of the imprinted mouse Rasgrf1 locus. Science 332, 848–852. 10.1126/science.1203919 21566194 PMC3368507

[B115] Wei W. Gilbert N. Ooi S. L. Lawler J. F. Ostertag E. M. Kazazian H. H.. (2001). Human L1 retrotransposition: cis preference versus trans complementation. Mol. Cell. Biol. 21, 1429–1439. 10.1128/MCB.21.4.1429-1439.2001 11158327 PMC99594

[B116] Woolfe A. Goodson M. Goode D. K. Snell P. McEwen G. K. Vavouri T.. (2005). Highly conserved non-coding sequences are associated with vertebrate development. PLoS Biol. 3:e7. 10.1371/journal.pbio.0030007 15630479 PMC526512

[B117] Yang N. Kazazian H. H. Jr. (2006). L1 retrotransposition is suppressed by endogenously encoded small interfering RNAs in human cultured cells. Nat. Struc. Mol. Biol. 13, 763–771. 10.1038/nsmb1141 16936727

[B118] Zhang H. Chen Z. Wang X. Huang Z. He Z. Chen Y. (2013). Long non-coding RNA: a new player in cancer. J. Hemat. Oncol. 6:37. 10.1186/1756-8722-6-37 23725405 PMC3693878

